# Rutin and *Moringa oleifera* leaf extract prevent monosodium glutamate-induced testicular toxicity in adult male albino rats

**DOI:** 10.3389/fvets.2025.1566471

**Published:** 2025-05-09

**Authors:** Doaa M. Abdel-Aty, Mona A. Ibrahim, Sherif R. Mohamed, Manal F. El-Khadragy, Ahmed E. Abdel Moneim, Ayah S. Fathalla, Doaa Soliman

**Affiliations:** ^1^Department of Zoology and Entomology, Faculty of Science, Helwan University, Cairo, Egypt; ^2^Department of Biology, College of Science, Princess Nourah Bint Abdulrahman University, Riyadh, Saudi Arabia; ^3^Al-Ayen Scientific Research Center, Al-Ayen Iraqi University, AUIQ, Nasiriyah, Iraq

**Keywords:** antioxidants, male reproductive system, oxidative stress, inflammation, histology, histochemistry, immunohistochemistry

## Abstract

**Introduction:**

Monosodium glutamate (MG) is a substance often used to enhance food flavor, but its effect on the reproductive system is known to have a negative impact. This study assessed the protective effects of rutin (RUT) and *Moringa oleifera* leaf extract (MOLE) on testicular toxicity induced by MG in rats.

**Methods:**

There were six groups: Control, RUT, MOLE, MG, RUT + MG, and MOLE + MG. The critical parameters measured were testicular index, hormone levels, antioxidants, oxidative stress markers, inflammation, apoptosis and histopathological changes.

**Results:**

Following MG exposure (60 mg/kg/day for 30 days), the testicular index and serum testosterone, LH, and FSH levels were significantly reduced. The markers of oxidative stress increased, whereas the antioxidants decreased. The levels of inflammatory and apoptotic markers increased. The increased expression of inflammatory and apoptotic markers and significant testicular tissue damage, including degenerative changes in the seminiferous tubules, infiltration of inflammatory cells, and deposition of collagen fibers were investigated in addition to an increase in inflammatory and apoptotic markers.

**Discussion:**

The present study showed that pre-administration of RUT or MOLE ameliorated the deleterious effects of MG, possibly due to antioxidant and anti-inflammatory properties, indicating a protective effect of RUT and MOLE on MG-induced testicular toxicity.

## 1 Introduction

Monosodium glutamate (MG), often known as sodium glutamate, is the sodium salt of L-glutamic acid, an amino acid (1, 2). MG comprises 78% glutamic acid, 22% sodium, and water (1). MG is a common culinary flavoring agent (3). It is often used to increase the umami flavor in the food business, particularly in the creation of processed and packaged goods as well as in restaurant meals and snacks. MG increases the overall flavor profile of foods by stimulating taste receptors on the tongue (4). The MG market was valued at USD 4.95 billion in 2022 and is projected to see over 5.7% Compound Annual Growth Rate (CAGR) between 2023 and 2032. Driven by the expanding customer preferences for flavor enhancers in the food and beverage industries (5).

The recommended quantity of added glutamate to increase food flavor should be between 0.1 and 0.8% of the product’s weight, akin to the natural L-glutamate concentration in tomatoes or Parmesan cheese. Excessive glutamate levels might severely impact flavor (6). Given the food industry’s high need for this additive, MG has been related to many health problems because its administration might raise the frequency of pachytene stage cells in primary spermatocytes (4), produce oxidative stress (OS) (7), and create free radicals (8). Additionally, MG use has been connected to obesity (9), central nervous system problems (10), reproductive abnormalities (11), hepatic damage (12), and hypertension (13). The testis is the primary organ of reproduction and endocrinal activities in males. It is very susceptible to injury due to its highly proliferating nature (14). The mechanism of action of MG-induced damage to diverse organs such as the liver, brain, testis, and kidney is connected to the production of oxidative stress (15) and inflammatory response (16).

Rutin (RUT), or rutinoside or quercetin-3-O-rutin, is a prevalent flavonoid occurring extensively in many vegetables and fruits (17, 18). This glycoside flavonoid is prevalent in orange peels and tomatoes and is particularly prominent in sophora rice and buckwheat flowers (19, 20). Rutin has shown the power to scavenge free radicals and alter critical signaling pathways such as Nrf2, MAPK, and NF-κB, which are implicated in treating certain disorders (21). Studies have indicated that RUT exhibits diverse pharmacological properties, such as antibacterial, hepatoprotective, immunomodulatory, antioxidant, and estrogenic actions (22). Moreover, investigations have demonstrated that RUT mitigates cisplatin-induced kidney damage and cell death in Wistar rats by reducing tumor necrosis factor-alpha (TNF-*α*), nuclear factor-kappa B (NF-κB), and caspase-3 mRNA expression levels (23). Kandemir et al. (24) discovered that RUT alleviates apoptosis and oxidative stress, lowering gentamicin-induced nephrotoxicity. This protective effect was coupled with activating antioxidant enzymes, mainly superoxide dismutase and glutathione peroxidase, and decreased lipid peroxidation levels in the testicular tissues (25).

*Moringa oleifera* (MO) is a fast-growing and drought-tolerant tropical plant. It is a member of the Moringaceae family. It has many names, including miracle, horseradish, benzalum, and drumstick trees (26). It grows in all tropical and subtropical areas such as Pakistan, Arabia, the sub-Himalayan regions of India, Central America, and the northern and southern Philippines (27). It is famous for increasing its biologically active compounds, principally in its leaves, which are broadly important for their nutritional and medicinal advantages (28, 29). The leaves contain vitamins, polyphenols, phenolic acids, flavonoids, alkaloids, carotenoids, glucosinolates, isothiocyanates, saponins, and tannins (30). These combinations have proved effective as antioxidants, anti-carcinogenic, and antimicrobial agents (19). MO leaves additionally provide a safeguard against oxidative stress (20), inflammation (31), bacterial action (32), fibrosis of the liver (33), damage of the liver (34), and hypercholesterolemia (35).

*In vivo* and *in vitro* research has been done to study the effects of MO leaves on the male reproductive system (36, 37). These findings emphasize that MO can help decrease numerous risk factors connected with male infertility. MO leaves, abundant in antioxidants and cytoprotective natural compounds, present it as a viable future technique in alleviating anomalies connected to cellular peroxidative damage and apoptosis (38). The amelioration found in testicular abnormalities, poor spermatogenesis, disturbances in the reproductive system, redox imbalances, and increased cell death (39, 40) post-supplementation with MO extract emphasizes their potential in addressing reproductive problems.

This study aimed to investigate the potential protective effects of RUT and MO leaf extract on the gonads of male rats exposed to MG and to explore the mechanisms underlying these effects.

## 2 Materials and methods

### 2.1 Chemicals

Monosodium glutamate with 99% purity was acquired online from Morgan Chemical Industry Company in Egypt, while rutin powder (non-citrus source; *Sophora japonica*; Flower Bud) was obtained from Sigma Company in Egypt.

### 2.2 Collection and extraction of plant

Fresh leaves of *Moringa oleifera* were collected from Helwan, Cairo, Egypt, during the summer, then identified and authenticated at the National Research Center in Giza. After cleaning, the leaves were air-dried and ground into powder. An aqueous extract was prepared by mixing 40 g of the dry powder with 100 milliliters (mL) of heated water for 1 h, stirring frequently. The mixture was then filtered using Whatman No. 1 paper at 55°C, and the filtrate was concentrated to 8% of its original volume using an RE-2010 rotary evaporator (BIOBASE, China) (41).

### 2.3 Phytochemical analysis of MOLE

Using an Interspec 200-X FTIR spectrophotometer (Spectronic Camspec Ltd., United Kingdom), MOLE was subjected to Fourier transform infrared (FTIR) spectroscopic analysis. The transmittance was scanned in the mid-IR region, ranging from 4,000 to 400 cm^−1^.

### 2.4 Animals and experimental protocol

Mature male Wistar albino rats (120–150 g, 8 weeks old) were obtained from the Medical Ain-Shams Research Institute in Cairo, Egypt. The experiment adhered to the Ain-Shams Research Institute Animal Facility guidelines under veterinary supervision. The protocol was conducted following the ethical research guidelines of Ain-Shams University, with the Experimental Animal Research Unit code number [RE (189)22]. The rats were housed under standard conditions, with a 12-h light/dark cycle, a temperature of 24 ± 2°C, and humidity of 50 ± 10%. They were allowed to acclimate for 1 week before the experiment began.

To investigate the protective effects of rutin and *Moringa oleifera* leaf extract on monosodium glutamate-induced reproductive toxicity, 60 rats were divided into six equal groups (*n* = 10 each) as follows:

Group 1—Control (CONT) group: Rats received no treatment.
Group 2—Rutin (RUT) group: Rats were given a daily dose of 150 mg/kg of rutin for 30 days (23).
Group 3**—***Moringa oleifera* leaf extract (MOLE) group: Rats received a daily dose of 500 mg/kg of *Moringa oleifera* leaf extract for 30 days (42).
Group 4—Monosodium glutamate (MG) group: Rats were administered an oral daily dose of 60 mg/kg of monosodium glutamate for 30 days (43). The lethal dose 50 (LD_50_) of MG in Wistar rats has been stated to be 500 mg/kg body weight, according to the study described by Airaodion et al. (44). The selected dose of our study (60 mg/kg body weight) is significantly lower than the reported LD_50_, representing only 12% of the lethal dose. This indicates that the chosen dose is within a non-lethal range.
Group 5 and 6—RUT + MG and MOLE + MG groups: Rats received rutin and *Moringa oleifera* leaf extract, respectively, at the same doses as their groups, followed by MG at the same dose 1 h later for 30 days.

### 2.5 Sample collection

At the end of the experiment, the rats were anesthetized for euthanasia by 1.9% inhaled diethyl ether (0.08 mL/liter of container volume) (45). They were exposed to ether for approximately 2 min in a transparent acrylic jar. After anesthesia, blood samples were collected from the retro-orbital sinus using a microhematocrit capillary tube, draining 2 mL of blood. The collected blood was placed in sterile labeled tubes (Serum Separator Clot Activator with Gel) and centrifuged (3,000 rpm/15 min.) to obtain serum, which was then stored at −20°C for the determination of testosterone, LH, and FSH levels. Testes from each group were carefully dissected, weighed, and divided into two sections. One section was fixed for 24 h. at room temperature in 10% neutral buffered formalin for histological, histochemical, and immunohistochemical investigations. The other section was stored at −80°C until ground and homogenized with cold 50 mM Tris–HCl buffer (pH 7.4). The homogenate (10% w/v) was then centrifuged at 3,000 × g for 10 min at 4°C to obtain the supernatant. This supernatant was kept at −20°C for the biochemical evaluation of oxidative stress, antioxidant, inflammatory, and apoptotic biomarkers.

### 2.6 Testicular index

The final body and testicular weights were measured with a precise weighing balance (Radwag, Model AS220/C/2, Clarkson Laboratory and Supply Inc., Chula Vista, CA, United States). The relative testicular weight was then calculated using the following formula:


$$\mathrm{Relative}\;\mathrm{testicular}\;\mathrm{weight}\;\left(RTW\right)=\frac{\mathrm{Left}\&\mathrm{right}\;\mathrm{testes}\;\mathrm{weight}\;}{\mathrm{Final}\;\mathrm{body}\;\mathrm{weight}}\times 100$$

### 2.7 Hormonal analyses

The levels of serum testosterone, luteinizing hormone (LH), and follicle-stimulating hormone (FSH) were quantitatively measured using the ELISA technique. Rat-specific kits purchased from BioVendor (Gunma, Japan) were used following the protocol provided with each kit.

### 2.8 Oxidative stress markers in the testicular tissue

Using testicular homogenate, the lipid peroxide (LPO) level was estimated by measuring the concentration of malondialdehyde (MDA), an end product of lipid peroxidation, according to the method described by Ohkawa et al. (46). The nitric oxide (NO) level was detected using the Griess reagent, following the method outlined by Green et al. (47). The reduced glutathione (GSH) concentration was determined using the method developed by Ellman (48).

### 2.9 Antioxidant status in the testicular tissue

The activity of testicular superoxide dismutase (SOD) was assessed using the standard method described by Nishikimi et al. (49). Testicular catalase (CAT) activity was measured following the technique outlined by Aebi (50). The activities of glutathione peroxidase (GPx) and glutathione reductase (GR) in testicular tissue were determined according to the methods described by Paglia and Valentine (51) and De Vega et al. (52), respectively.

### 2.10 Inflammatory marker assays

Testicular levels of the pro-inflammatory cytokines tumor necrosis factor-alpha (TNF-*α*) and interleukin-1 beta (IL-1β) were measured using ELISA kits obtained from R&D Systems and Thermo Fisher Scientific, respectively, following the manufacturer’s protocols.

### 2.11 Apoptotic marker assays

The levels of Bcl-2, Bax, and caspase-3 proteins in testicular tissues were assessed using commercial ELISA kits obtained from BioVision, Inc. (Bcl-2: Cusabio, Catalog # CSB-E08854r; Bax: BioVision, Inc., Catalog # E4513; caspase-3: Sigma-Aldrich, Catalog # CASP3C-1KT), following the manufacturer’s instructions.

### 2.12 Histopathological examinations

The fixed testes were prepared for paraffin sectioning, which included dehydration, clearing, infiltration, and embedding in paraffin wax. Sections with a thickness of 5 microns were cut using a rotary microtome, stained for histological, histochemical, and immunohistochemical investigations, examined under a light microscope (LEICA DM4P, Wetzlar, Germany), and photographed using a Zeiss camera.

### 2.13 Histological and histochemical results

For the general histopathological study of the tissue, we used Hematoxylin and Eosin (H&E) stain (53), and Masson’s Trichrome stain for demonstration of the tissue collagen fibers (54). Periodic acid-Schiff (PAS) histochemical technique was utilized to detect polysaccharides in tissue (55).

### 2.14 Immunohistochemical results

The proliferating cell nuclear antigen (PCNA) acts as a marker for cell proliferation (56), while caspase-3 indicates apoptosis (57), and cyclooxygenase-2 (COX-2) is primarily linked with inflammation (58). Sections of testicular tissue from each experimental group were subjected to staining using anti-PCNA, anti-Caspase-3, and anti-COX-2 antibodies. The avidin-biotin-peroxidase technique detected PCNA, caspase-3, and COX-2 immunoreactivities in the testis (59).

### 2.15 Morphometric analysis

Histomorphometric analysis determined the mean area percentage of collagen fibers, polysaccharides, PCNA, Caspase-3, and COX-2 positive immunoreactions. This measurement was taken in 10 non-overlapping high-power fields (40×) of paraffin sections of the testis for each group, utilizing ImageJ software (version 1.46, NIH, United States) and subsequently analyzed statistically.

### 2.16 Statistical analysis

Data from various evaluations were analyzed using one-way analysis of variance (ANOVA) and Tukey’s multiple comparisons test, utilizing GraphPad Prism software (version 8.00). A *p*-value of <0.05 was considered statistically significant. The analyzed data are presented as the mean ± SD.

## 3 Results

### 3.1 Phytochemical analysis of MOLE

**Figure 1:**
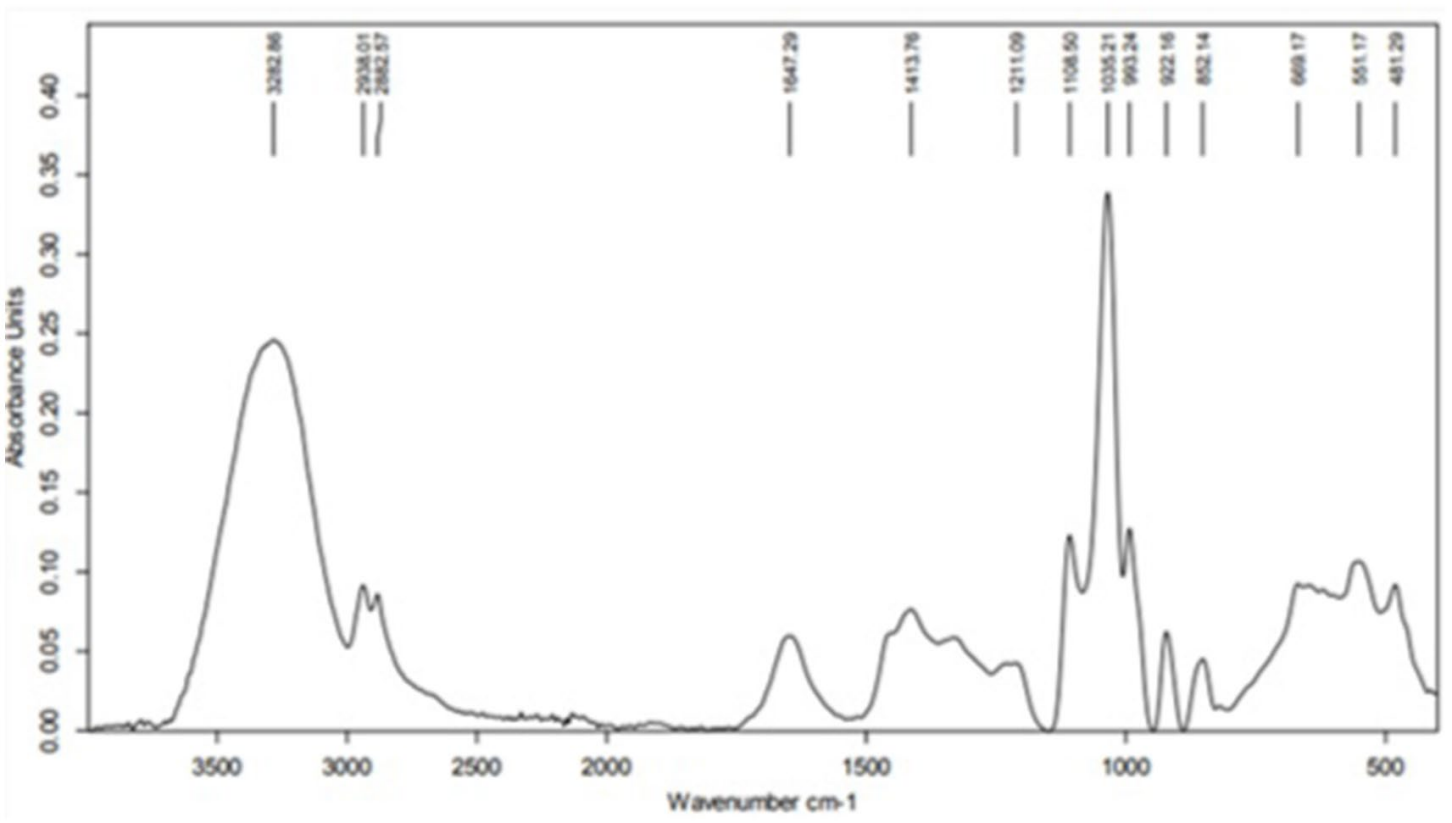
Fourier-transform infrared spectroscopy spectrum of the crude aqueous *Moringa oleifera* leaf extract.

The Fourier Transform Infrared (FTIR) spectrum of the crude aqueous MOLE represented in Figure 1 provided information on the functional groups present in the sample. A detailed analysis of the peaks in the spectrum:

#### 3.1.1 High wavenumber region (4,000–2,500 cm^−1^)

1. 3282.86 cm^−1^: This broad peak is characteristic of O-H stretching vibrations, commonly associated with hydroxyl groups in alcohols, phenols, and water. The broad nature of the peak indicates hydrogen bonding, typical of water or phenolic compounds.
2. 2923.01 cm^−1^ and 2852.57 cm^−1^: These peaks correspond to the asymmetric and symmetric stretching vibrations of C-H bonds in alkanes, indicating the presence of methylene (-CH₂) groups, which are typical in fatty acids and lipids.

#### 3.1.2 Mid wavenumber region (2,500–1,500 cm^−1^)

1. 31647.29 cm^−1^: This peak likely represents the C=O stretching vibration, typically found in carbonyl groups such as ketones, aldehydes, carboxylic acids, or esters. It could also indicate the presence of conjugated alkenes or amides.
2. 41413.76 cm^−1^: This peak is indicative of C-H bending (scissoring) vibrations, which are characteristic of alkanes.
3. 51211.09 cm^−1^: This peak could be associated with C-O stretching vibrations, often seen in ethers, alcohols, and esters.

#### 3.1.3 Low wavenumber region (1,500–500 cm^−1^)

1. 1108.50 cm^−1^ and 1033.21 cm^−1^: These peaks are associated with C-O stretching vibrations, typical of alcohols, esters, or carboxylic acids. They may also indicate the presence of polysaccharides or glycosidic linkages.
2. 922.16 cm^−1^, 852.14 cm^−1^: These peaks likely represent out-of-plane bending vibrations of = C-H bonds, indicating the presence of alkenes or aromatic compounds.
3. 668.17 cm^−1^ and 555.17 cm^−1^: These peaks correspond to bending vibrations of functional groups, which could be associated with alkyl halides or other substituents.
4. 481.29 cm^−1^: This low-frequency peak might correspond to skeletal vibrations or bending vibrations in larger molecular structures, possibly indicating inorganic compounds or metal-oxygen bonds.

### 3.2 Testicular index

**Figure 2:**
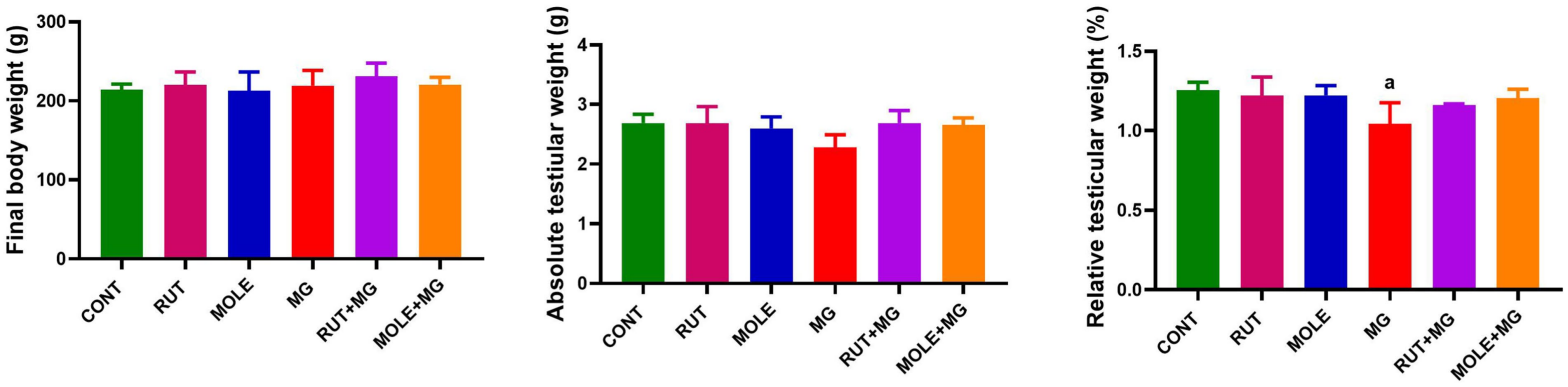
Effects of rutin (RUT) and *Moringa oleifera* leaf extract (MOLE) pre-administration on final body weight and absolute and relative testicular weight in rats treated with monosodium glutamate (MG). All values are stated as mean ± SD (*n* = 10). ^a^Points to a significant change vs. the control (CONT) group at *p* < 0.05, using Tukey’s multiple comparisons test.

The effect of MG on final body weights absolute and relative testicular weights was represented in Figure 2. Compared with the standard control group, no marked differences were found between the final body weights of all groups. The administration of MG to rats caused a slight decrease in absolute testicular weight and a significant decrease in relative testicular weight compared to the CONT group. Pre-administration of RUT or MOLE to MG did not change the absolute and relative testes weight compared to the control group.

### 3.3 Hormonal analyses

**Figure 3:**
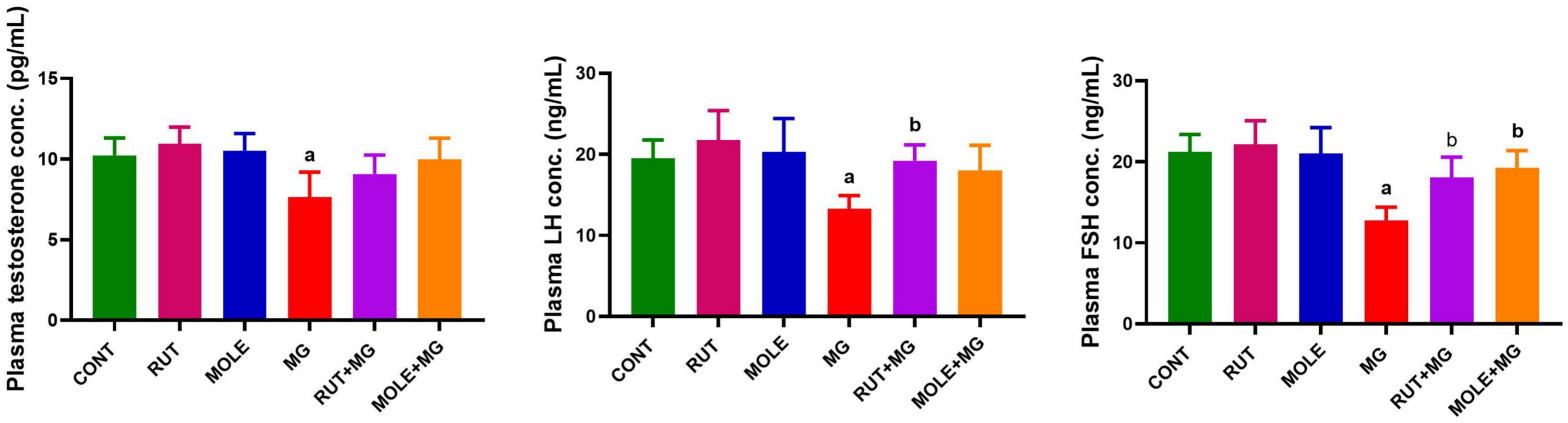
Effects of RUT and MOLE pre-administration on serological testosterone, luteinizing hormone (LH), and follicle-stimulating hormone (FSH) levels in rats exposed to MG. All values are stated as mean ± SD (*n* = 10). ^a^Points to a significant change vs. the CONT group at *p* < 0.05; ^b^points to a significant change vs. the MG group at *p* < 0.05, using Tukey’s multiple comparisons test.

The administration of MG reduced serum testosterone, LH, and FSH levels compared to the CONT group. The pre-administration of RUT or MOLE to MG increased testosterone, LH, and FSH levels compared to MG, indicating a protective effect of RUT and MOLE against MG-induced reduction in testosterone, LH, and FSH (Figure 3).

### 3.4 Oxidative stress markers in the testicular tissue

**Figure 4:**
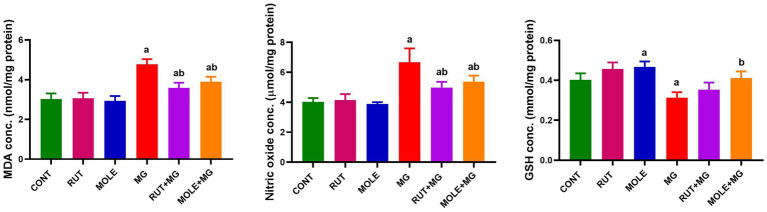
Effects of RUT and MOLE pre-administration on malondialdehyde (MDA), nitric oxide, and reduced glutathione (GSH) levels in the testes of rats treated with MG. Values of oxidative stress markers’ levels are stated as mean ± SD (*n* = 10). ^a^Points to a significant change vs. the CONT group at *p* < 0.05; ^b^points to a significant change vs. the MG group at *p* < 0.05, using Tukey’s multiple comparisons tests.

Administration of MG led to a significant increase in lipid peroxidation, as evidenced by elevated MDA levels and higher NO levels while causing a decrease in GSH levels in the testicular tissues compared to the CONT group. The pre-administration of RUT or MOLE significantly mitigated the oxidative stress markers’ levels in the testes of rats treated with MG. Both RUT and MOLE reduced MDA and nitric oxide levels while increasing GSH levels compared to the MG group, indicating their protective effects against oxidative stress induced by MG treatment. However, the levels of these markers ultimately did not return to the baseline levels observed in the CONT group (Figure 4).

### 3.5 Antioxidant status in the testicular tissue

**Figure 5:**
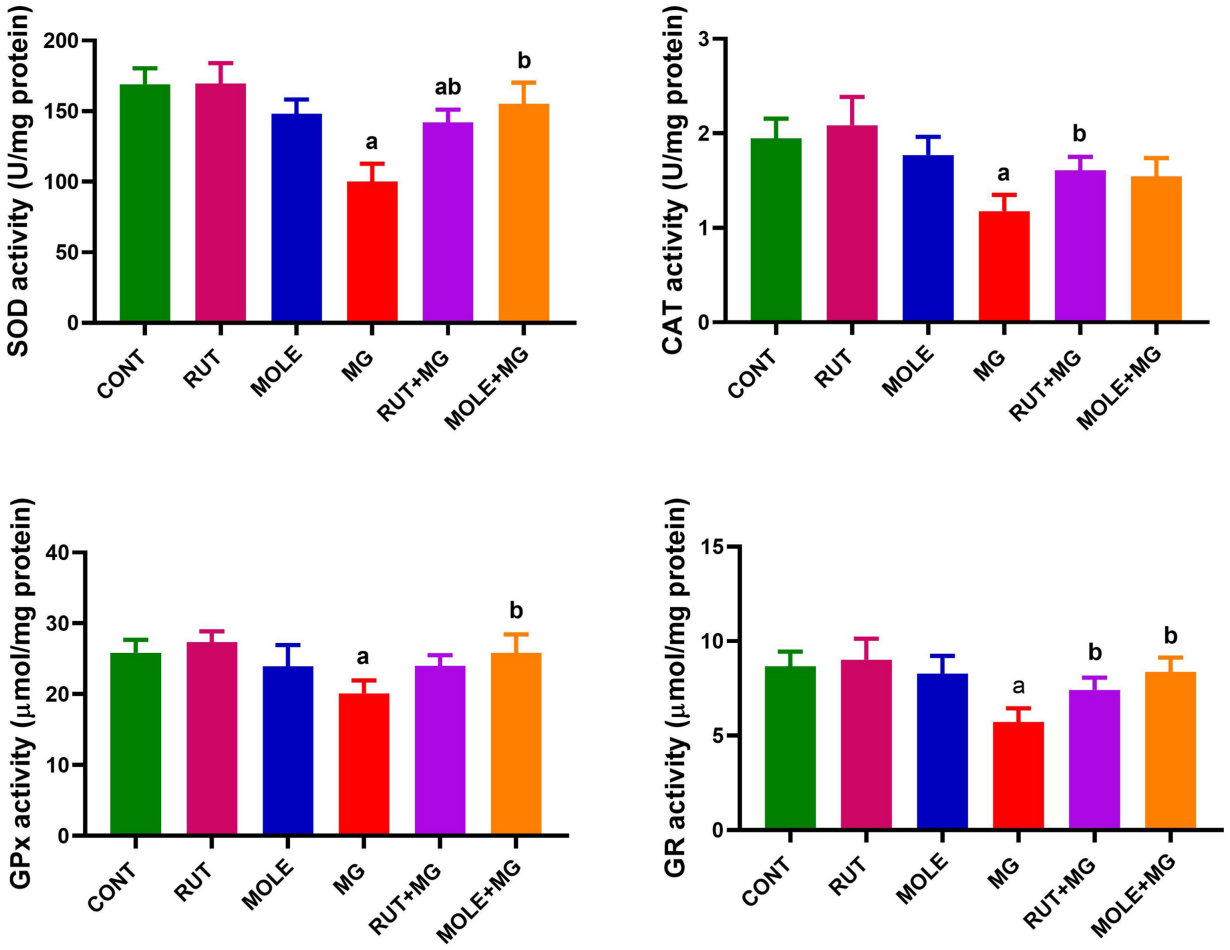
Effects of RUT and MOLE pre-administration on the levels of superoxide dismutase (SOD), catalase (CAT), glutathione peroxidase (GPx), glutathione reductase (GR) in the testes of rats treated with MG. Values of antioxidant enzyme activities are expressed as the mean ± SD (*n* = 10). ^a^Points to a significant change vs. the CONT group at *p* < 0.05; ^b^points to a significant change vs. the MG group at *p* < 0.05, using Tukey’s multiple comparisons test.

In MG-treated rats, the activity of all antioxidant enzymes (SOD, CAT, GPx, and GR) was significantly reduced when compared to the control group at *p* < 0.05. However, the SOD activity remains slightly decreased in the RUT + MG group compared to the control group; the pre-administration of RUT or MOLE to MG significantly improved the activities of SOD, CAT, GPx, and GR in the testes of rats treated with MG, counteracting the adverse effects of MG consumption (Figure 5).

### 3.6 Inflammatory marker assays

**Figure 6:**
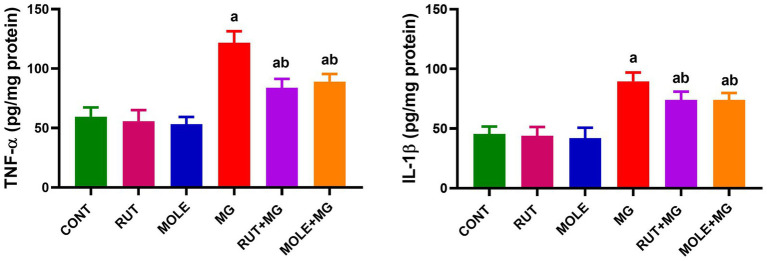
Effects of RUT and MOLE pre-administration on the levels of tumor necrosis factor-*α* (TNF-α) and interleukin-1β (IL-1β) in the testes of rats treated with MG. All values are stated as mean ± SD (*n* = 10). ^a^Points to a significant change vs. the CONT group at *p* < 0.05; ^b^points to a significant change vs. the MG group at *p* < 0.05, using Tukey’s multiple comparisons test.

In MG-treated rats, the levels of inflammatory markers (TNF-*α* and IL-1β) were significantly increased compared to the control group at *p* < 0.05. The pre-administration of RUT or MOLE significantly reduced TNF-α and IL-1β levels in the testes of rats treated with MG. These findings indicated that both RUT and MOLE had protective effects against inflammation induced by MG treatment. Pre-administration of these compounds to MG mitigated the elevation of TNF-α and IL-1β caused by MG, but the levels remained slightly elevated compared to the control group (Figure 6).

### 3.7 Apoptotic marker assays

**Figure 7:**
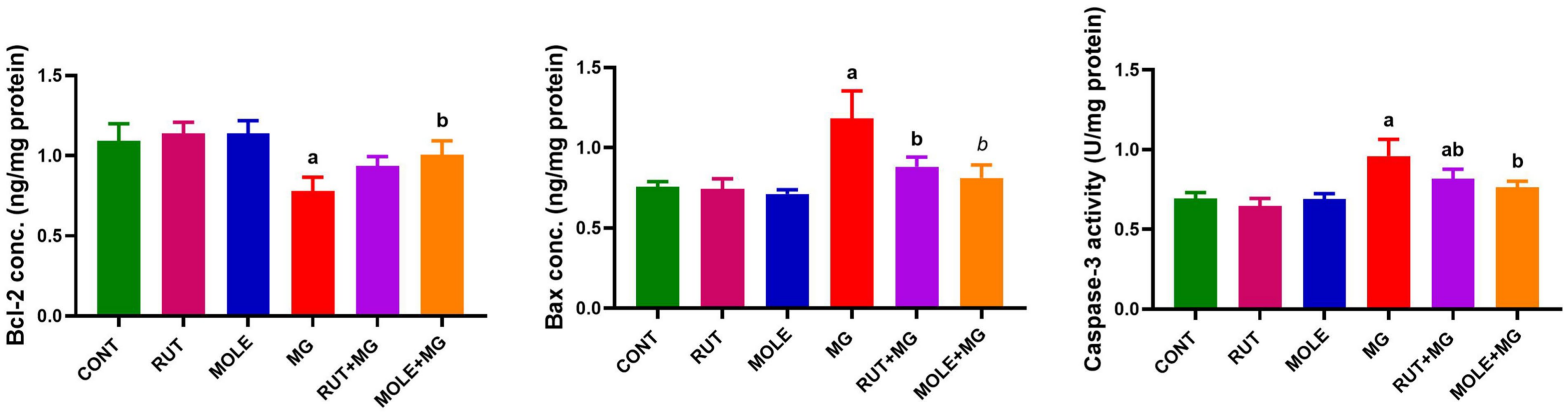
Effects of RUT and MOLE pre-administration on the levels of apoptotic proteins (Bcl-2, Bax and caspases-3) in the testes of rats treated with MG. All values are stated as mean ± SD (*n* = 10). ^a^Points to a significant change vs. the CONT group at *p* < 0.05; ^b^points to a significant change vs. the MG group at *p* < 0.05, using Tukey’s multiple comparisons test.

MG treatment increased apoptotic protein levels (Bax and caspase-3) and decreased anti-apoptotic protein levels (Bcl-2) in the testes of rats. However, the caspase-3 level remained slightly elevated in the RUT + MG group compared to the control group; the pre-administration of RUT or MOLE to MG counteracted the effects of MG, resulting in increased levels of Bcl-2 and decreased levels of Bax and caspase-3, suggesting a protective effect against MG-induced apoptosis (Figure 7).

### 3.8 Histological and histochemical results

#### 3.8.1 Hematoxylin and eosin staining

**Figure 8:**
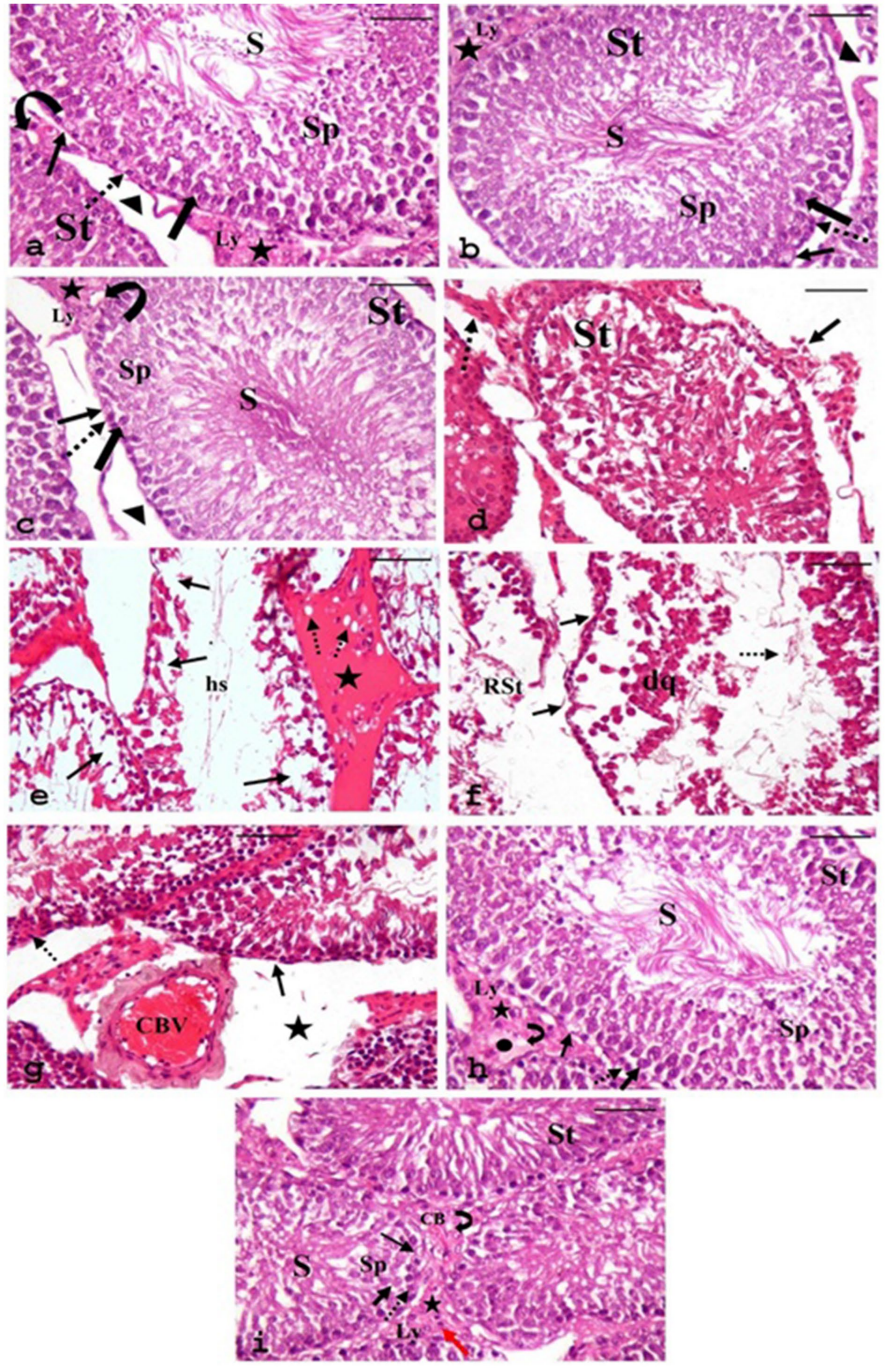
Photomicrographs of H&E-stained sections of testes from various groups. **(a)** CONT, **(b)** RUT, and **(c)** MOLE groups display seminiferous tubules (ST) surrounded by a basement membrane and myoid cells (arrowhead); interstitial tissue (asterisks) with blood vessels (curved arrow) and in between, clusters of Leydig cells (Ly) are seen. Each tubule is lined with Sertoli cells (thin arrow) and spermatogenic cells: spermatogonia (dashed arrow), primary spermatocytes (thick arrow), spermatids (SP), and numerous spermatozoa (S) filling the lumen. Panels **(d–g)** depict the MG group: panel **(d)** shows irregular organization of germinal epithelium in the seminiferous tubule (St) with discrete interstitial tissue (arrow) and inflammatory infiltration in the interstitium (dashed arrow). Panel **(e)** reveals distorted seminiferous tubules with focal areas of spermatogenic cell loss (arrow), wide lumina without sperm, filled with acidophilic hyaline streaks (hs), and widened, vacuolated interstitium (dashed arrow) containing acidophilic hyaline material (star). Panel **(f)** demonstrates a ruptured seminiferous tubule (RST). Another tubule shows irregular basement membranes (arrow), detachment of spermatogenic cells from the basement membrane with desquamated cells (dq), and a few sperms (dashed arrow) within the tubular lumen. Panel **(g)** indicates congested blood vessels (CBV) in the interstitial tissue and the widening of interstitial spaces (star). Pyknosis (dashed arrow) and reduction in the height of the germinal epithelium (arrow) are evident. **(h,i)** RUT + MG and MOLE + MG groups show seminiferous tubules with active spermatogenesis and numerous sperm (S) in the lumen. The interstitial tissue (asterisks) appears nearly normal, with vacuolation (curved arrow) and edema (circle) in the RUT + MG group, while vacuolation (curved arrow), a few inflammatory cells (red arrow), and congestion of blood vessels (CB) are noted in the MOLE + MG group. Dashed arrow, Spermatogonia; thick arrow, Primary spermatocytes; SP, Spermatids; thin arrow, Sertoli cells; Ly, Leydig cells (scale bar = 50 μm).

Light microscopy examination of the H&E-stained sections of testes from the CONT group (Figure 8a) revealed the typical histo-architecture of the testis. The seminiferous tubules were separated by interstitial tissue containing blood vessels and clusters of Leydig cells, which exhibited pale acidophilic cytoplasm. A basement membrane and flat peritubular myoid cells bounded each tubule. These myoid cells resemble smooth muscle cells and are classified as myoepithelial cells. The seminiferous tubules were lined by stratified germinal epithelium consisting of spermatogenic cells and supportive Sertoli cells. Both cell types rested on a prominent basal lamina.

Sertoli cells were elongated, with large, basal, rounded, and pale nuclei. The spermatogenic cells were arranged in several layers. Spermatogonia, the small, rounded cells, rested on the basal lamina. Primary spermatocytes, the most significant and numerous rounded cells, were located above the spermatogonia. Spermatids, the smallest spermatogenic cells, were arranged in several rows near the tubular lumen. Spermatozoa were observed in the lumen of the seminiferous tubules.

The RUT and MOLE groups also displayed normal testicular histo-architecture (Figures 8b,c, respectively). In contrast, the sections from the MG group (Figures 8d–g) displayed numerous histological changes compared to the CONT group. The germinal epithelium of the seminiferous tubules exhibited irregular organization, with noticeable distortion of the seminiferous tubules and focal areas of spermatogenic cell loss. The lumina were expanded, devoid of sperm, and filled with acidophilic hyaline streaks. Some tubules exhibited irregular basement membranes and detachment of spermatogenic cells from the basement membrane, with desquamated cells and few sperm within the tubular lumen. Rupture of some seminiferous tubules was also observed. Pyknosis and a reduction in the height of the germinal epithelium of the seminiferous tubules were noted. The interstitium appeared widened, vacuolated, and filled with acidophilic hyaline material; leukocytic infiltration and congested blood vessels were also seen.

Compared to the MG group, sections from the RUT + MG group (Figure 8h) and the MOLE + MG group (Figure 8i) displayed fewer pathological changes. Most tubules exhibited typical structures with active spermatogenesis, and the lumens were filled with mature spermatozoa. However, the interstitial tissue between seminiferous tubules showed vacuolation and edema in the RUT + MG group, while the MOLE + MG group exhibited vacuolation, a few inflammatory cells and congestion of blood vessels.

#### 3.8.2 Masson’s trichrome staining

**Figure 9:**
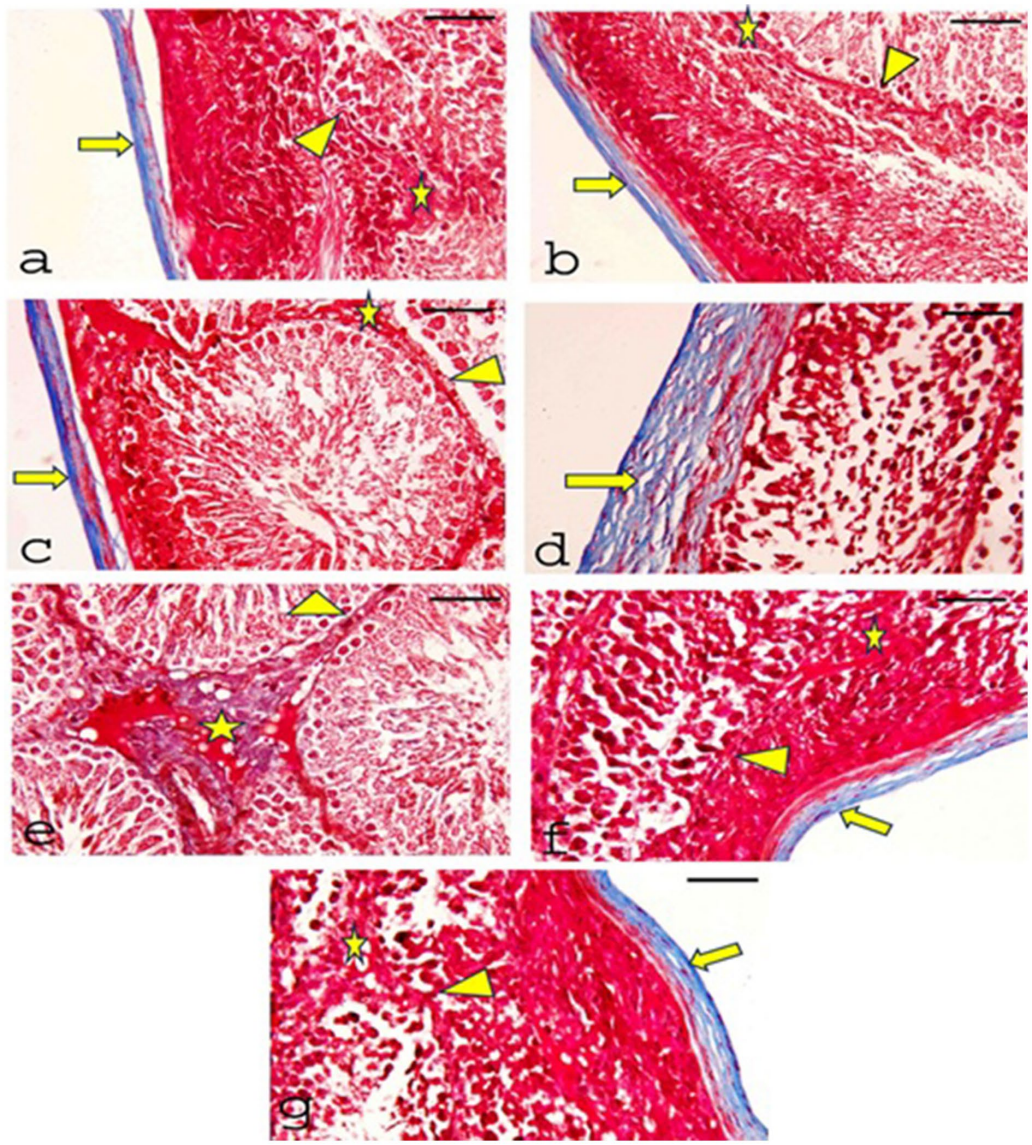
Photomicrographs of Masson’s Trichrome-stained sections of testes from all groups. **(a)** CONT, **(b)** RUT, and **(c)** MOLE groups show the tunica albuginea capsule formed of collagen fibers (blue) (arrow). There is no collagen fiber deposition in the interstitial tissues (asterisks) or the basement membranes of the tubules (arrowhead). **(d)** Moreover, **(e)** the MG group displays significant collagen fiber deposition in the capsule, which appears thickened and shows corrugation (arrow). Notable collagen fiber deposition is also observed in the interstitial tissue (asterisks) and basal lamina (arrowhead). **(f)** RUT + MG and **(g)** MOLE + MG groups exhibit a small number of collagen fibers in the capsule (arrow) but no collagen fibers in the interstitial tissue (asterisks) or the basal lamina of the seminiferous tubules (arrowhead) (scale bar = 50 μm).

Masson’s trichrome stained sections of testes demonstrated that the collagen fibers appeared blue, the CONT group (Figure 9a), RUT group (Figure 9b), and MOLE group (Figure 9c) showed that the tunica albuginea capsule was formed of collagen fibers and exhibited no deposition of collagen fibers in the interstitial tissues or the basement membranes of the tubules. In the MG group (Figures 9d,e), collagen fibers were corrugated in the thickened capsule, along with evident deposition of collagen fibers in the interstitial tissues and the basal lamina of the seminiferous tubules. Sections from the RUT+MG group (Figure 9f) and MOLE+MG group (Figure 9g) showed a capsule appearance similar to the control group. The tubules’ interstitial tissue and basal lamina did not display collagenous fibers.

#### 3.8.3 Periodic acid Schiff reaction “PAS”

**Figure 10:**
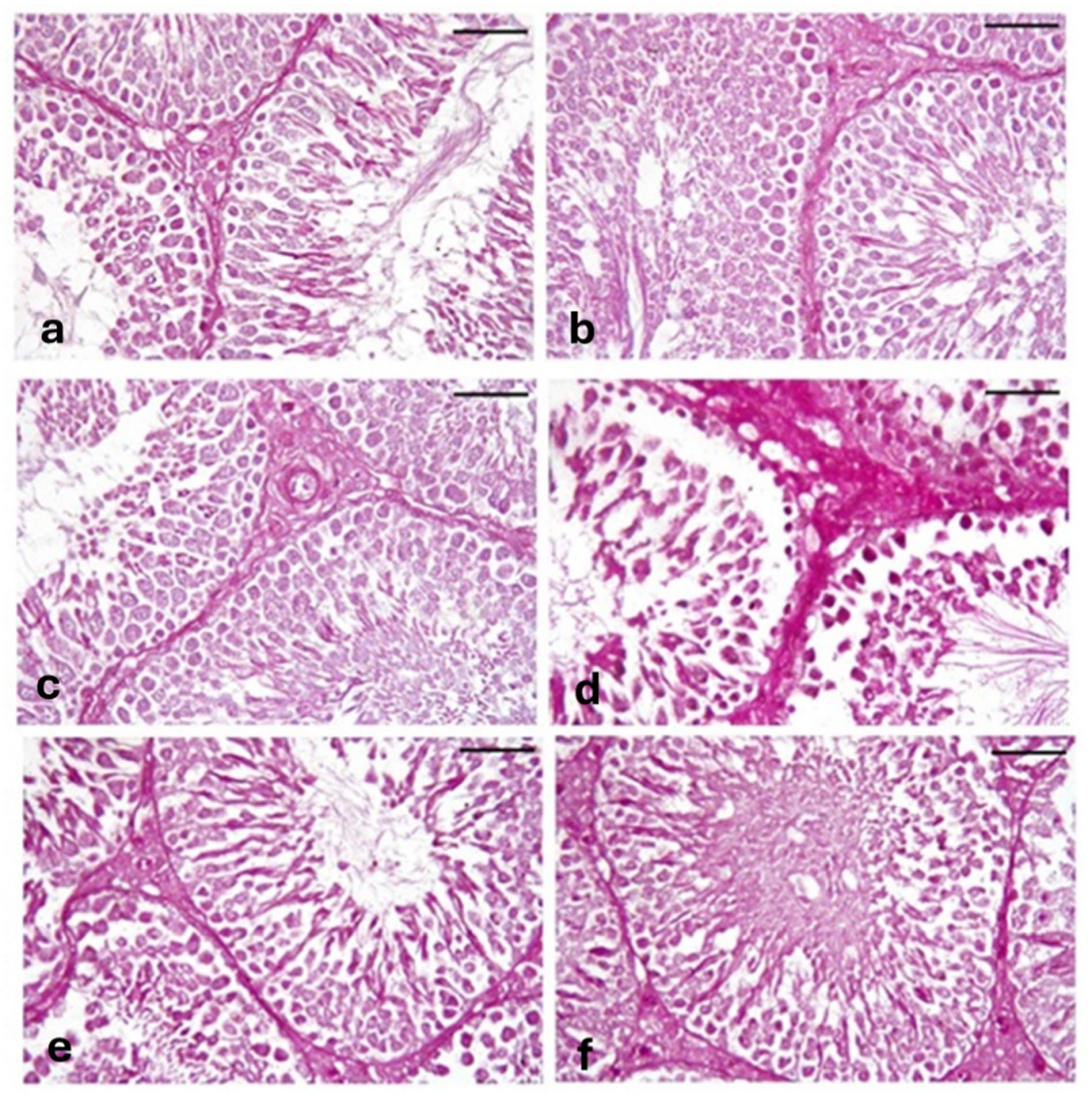
Photomicrographs of PAS-stained sections of testes across all groups. **(a)** CONT, **(b)** RUT, and **(c)** MOLE groups display a strong PAS reaction in the basal lamina of tubules, interstitial tissues, and spermatogenic cells. **(d)** The MG group exhibits an extreme PAS reaction in the basal lamina, interstitial tissues, and spermatogenic cells. **(e)** RUT + MG and **(f)** MOLE + MG groups show a reaction nearly similar to that of the control group (scale bar = 50 μm).

Sections of testes stained with PAS from the CONT group (Figure 10a), RUT group (Figure 10b), and MOLE group (Figure 10c) showed a strong PAS reaction in the basal lamina of tubules, interstitial tissues, and spermatogenic cells. The MG group (Figure 10d) exhibited a strong PAS reaction in the basal lamina, interstitial tissue, and spermatogenic cells. In contrast, sections from the RUT+MG (Figure 10e) and MOLE+MG (Figure 10f) groups displayed a reaction closely similar to that of the CONT group.

### 3.9 Immunohistochemical results

#### 3.9.1 Immunohistochemical staining for proliferating cell nuclear antigen

**Figure 11:**
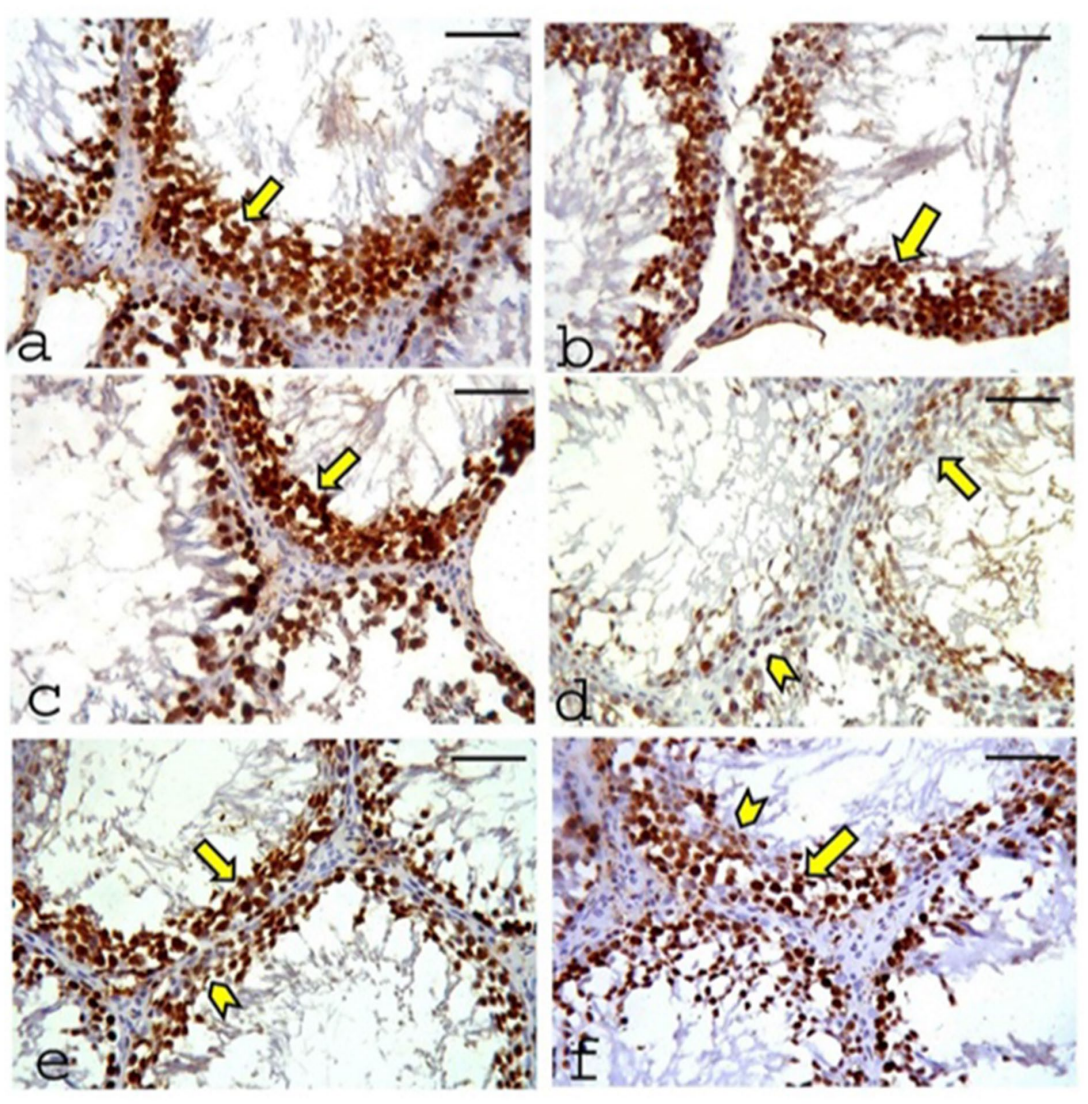
Photomicrographs of proliferating cell nuclear antigen (PCNA) expression in the testes of the studied groups. **(a)** CONT group, **(b)** RUT group, and **(c)** MOLE group show positive PCNA immunoreactivity (indicated by a deep brown reaction) in the nuclei of all germinal cells (arrow). **(d)** The MG group exhibits weak PCNA reactivity in most germinal cell nuclei (arrow), with only a few spermatogenic cells showing positive PCNA immunoreactivity (arrowhead). **(e)** RUT+MG and **(f)** MOLE+MG groups demonstrate positive PCNA immunoreactivity in most germ cell nuclei (arrow), although a few nuclei show a weak reaction (arrowhead) (scale bar = 50 μm).

A positive reaction for proliferating cell nuclear antigen (PCNA) is indicated by brown nuclear staining. The CONT (Figure 11a), RUT (Figure 11b), and MOLE (Figure 11c) groups exhibited positive PCNA immunoreactivity in all nuclei of the germinal cells. In contrast, sections from the MG group (Figure 11d) showed weak PCNA reactivity in most of the nuclei of the germinal cells, with only a few spermatogenic cells displaying positive PCNA immunoreactivity. Sections from the RUT+MG (Figure 11e) and MOLE+MG (Figure 11f) groups showed positive PCNA immunoreactivity in most nuclei of the germ cells, although a few nuclei displayed a weak reaction.

#### 3.9.2 Immunohistochemical staining for Cyclooxygenase-2

**Figure 12:**
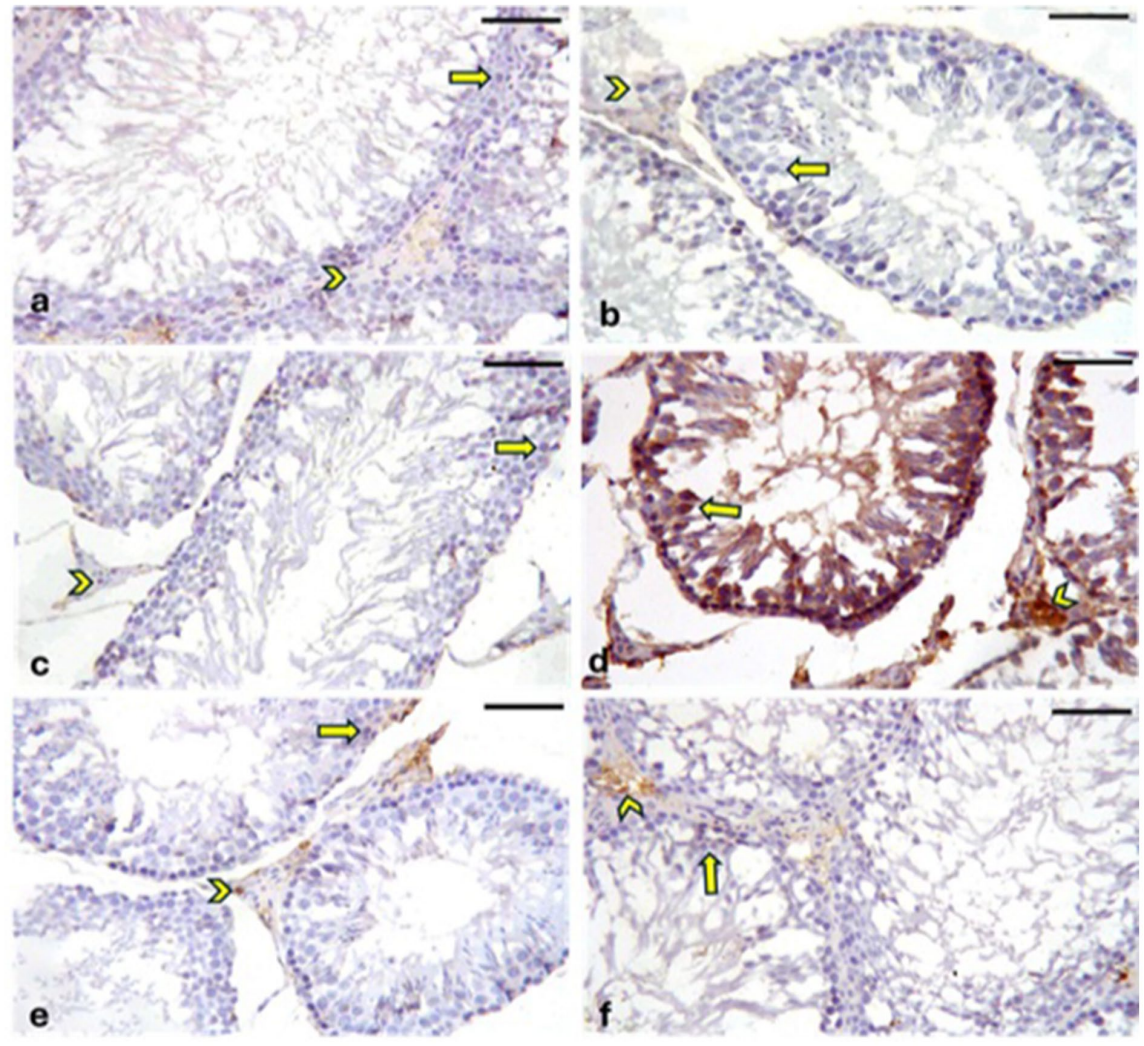
Photomicrographs showing Cyclooxygenase-2 (COX-2) expression in the testes across different groups. The CONT **(a)**, RUT **(b)**, and MOLE **(c)** groups exhibit negative to weak COX-2 immunoreaction in the cytoplasm or nucleus of the germinal epithelium (arrows) and interstitial Leydig cells (arrowheads). In contrast, the MG **(d)** group displays positive COX-2 immunoreactivity, indicated by a deep brown reaction, in the seminiferous epithelium (arrows) and interstitial Leydig cells (arrowheads). The RUT + MG **(e)** and MOLE + MG **(f)** groups show weak-to-moderate COX-2 immunoreaction in some seminiferous epithelia (arrows) and Leydig cells (arrowheads) (scale bar = 50 μm).

A positive reaction for Cyclooxygenase-2 (COX-2) is indicated by brown cytoplasmic or nuclear staining. Negative to weak cytoplasmic or nuclear COX-2 immunoreaction was observed in the germinal epithelia and interstitial Leydig cells of the CONT (Figure 12a), RUT (Figure 12b) and MOLE (Figure 12c) groups. In contrast, the MG group (Figure 12d) exhibited strong COX-2 immunoreactivity in the germinal epithelium and Leydig cells. The RUT + MG (Figure 12e) and MOLE + MG (Figure 12f) groups showed weak-to-moderate COX-2 immunoreaction in some seminiferous epithelia and Leydig cells.

#### 3.9.3 Immunohistochemical staining for caspase-3

**Figure 13:**
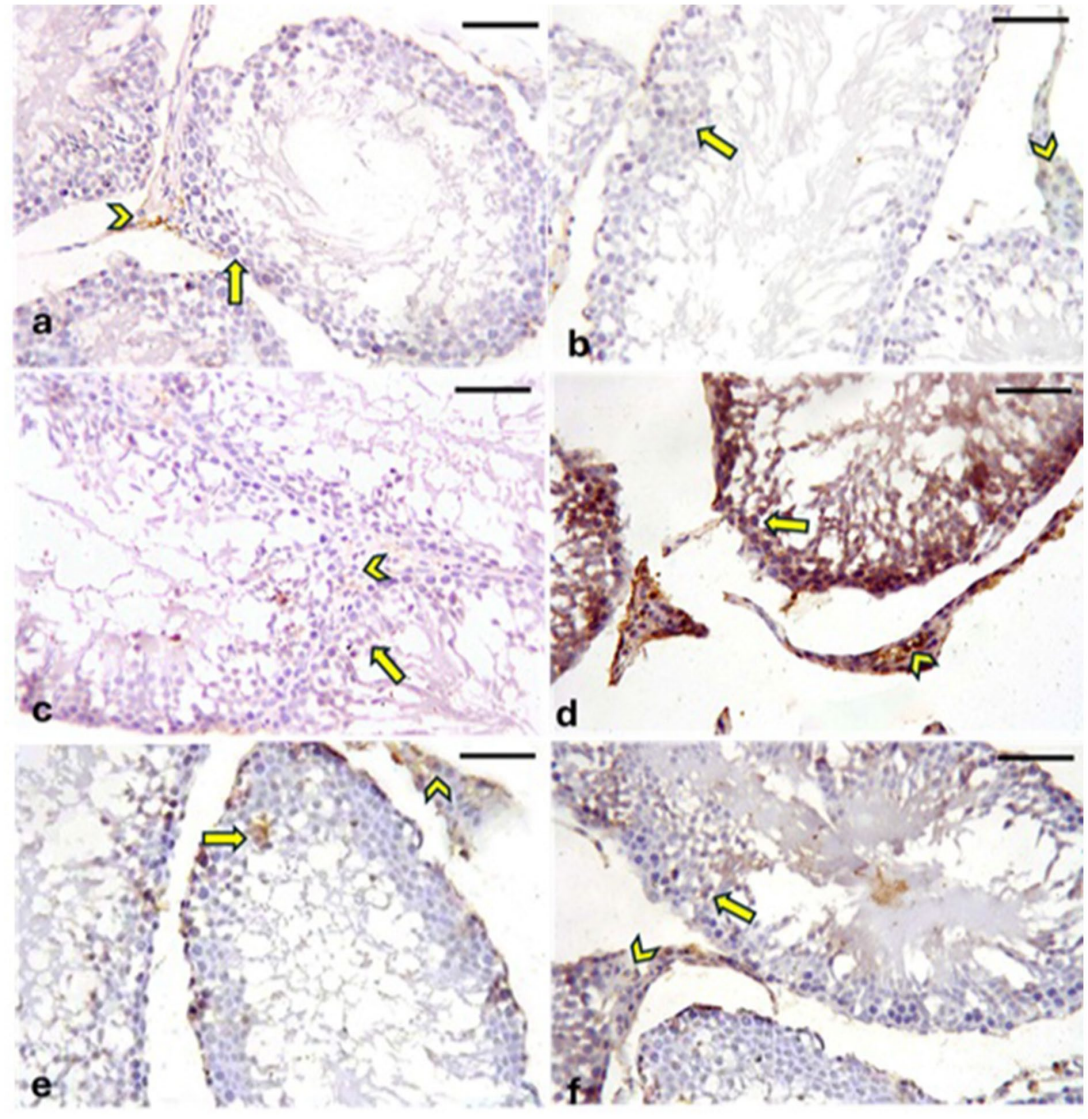
Photomicrographs showing caspase-3 expression in the testes across all groups. The CONT **(a)**, RUT **(b)**, and MOLE **(c)** groups display negative to weak caspase-3 immunoreactions in the germinal epithelia (arrows) and interstitial Leydig cells (arrowheads). In the MG group **(d)**, strong caspase-3 immunoreactivity (deep brown staining) is observed in both the seminiferous epithelia (arrows) and Leydig cells (arrowheads). The RUT + MG **(e)** and MOLE + MG **(f)** groups exhibit moderate caspase-3 immunoreactions in some seminiferous epithelia (arrows) and Leydig cells (arrowheads) (scale bar = 50 μm).

A positive reaction for *caspase-3* is indicated by brown cytoplasmic or nuclear staining. In the CONT (Figure 13a), RUT (Figure 13b), and MOLE (Figure 13c) groups, the immunoreaction of caspase-3 was either negative or weak in the cytoplasmic or nuclear regions of both the germinal epithelia and interstitial Leydig cells. Conversely, the MG group (Figure 13d) exhibited strong caspase-3 immunoreactivity in germinal epithelia and Leydig cells. The RUT + MG (Figure 13e) and MOLE + MG (Figure 13f) groups showed moderate caspase-3 immunoreaction in some germinal epithelia and Leydig cells.

### 3.10 Morphometric analysis

**Figure 14:**
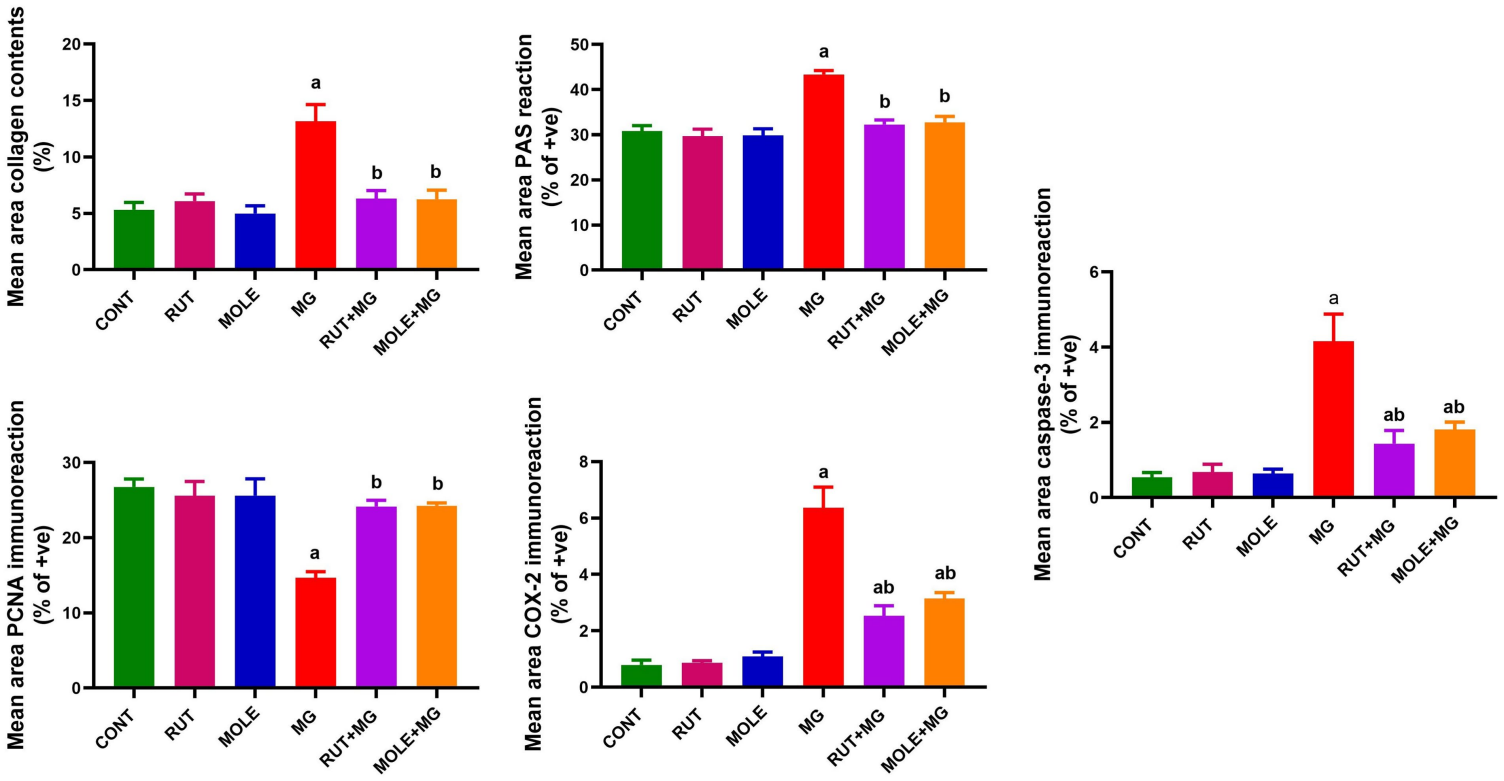
Quantitative analysis of mean area percentage of Masson’s trichrome-stained collagen contents, positive PAS reaction, and positive PCNA, COX2 and caspase-3 immunoreactions in the testicular tissues of different studied groups. Results are presented as mean ± SD. ^a^Indicates significant change vs. the control group at *p* < 0.05; ^b^indicates significant changes vs. the MG-treated group at *p* < 0.05.

The influence of RUT and MOLE on the mean area percentage values in the testicular tissue of rats intoxicated with MG was examined in Figure 14. The MG group showed a significant increase in the mean area percentage of collagen contents, PAS +ve reaction, COX2 and caspase-3 +ve immunoreaction, and a significant decrease in PCNA +ve immunoreaction compared to the CONT group at *p* < 0.05. pre-administration of rutin and MOLE to MG significantly increased the mean area percentage of collagen contents, PAS +ve reaction, COX2 and caspase-3 +ve immunoreaction and significantly decreased in PCNA +ve immunoreaction in testicular tissues compared to the MG group at *p* < 0.05. This impact indicated their protective effects against the damage induced by MG treatment, although the mean area percentage of COX2 and caspase-3 +ve immunoreaction levels did not return ultimately to the baseline levels observed in the CONT group.

## 4 Discussion

Monosodium glutamate is a prevalent food ingredient in numerous processed and seasoned foods. Although MG has been linked to numerous adverse consequences on biological functions, studies have specifically emphasized its deleterious influence on the male reproductive system. This study examined the possible protective effects of rutin and *Moringa oleifera* leaf extract against MG-induced testicular damage in male rats.

Our biochemical experiments demonstrated that MG treatment markedly raised oxidative stress, evidenced by higher levels of nitric oxide and MDA, a biomarker of lipid peroxidation. A significant reduction in GSH levels further emphasized compromised antioxidant defenses. Furthermore, diminished activities of critical antioxidant enzymes (SOD, CAT, GPx, and GR) in testicular tissues indicate that MG compromises the enzymatic antioxidant defense mechanism, exacerbating testicular injury.

Oxidative stress appears after an imbalance between the capacity of oxidation and antioxidation in the body that leads to the excessive manufacture of reactive molecules like reactive nitrogen species (RNS) and reactive oxygen species (ROS). These unsteady radicals, involving hydrogen peroxide (H_2_O_2_), hydroxyl radicals (OH^−^), nitric oxide (NO), and superoxide anion (O^2−^), can produce significant tissue damage (60).

Lipid peroxidation, driven by ROS, results in the generation of lipid hydroperoxides (LOOHs) as primary products and secondary aldehyde products, including MDA, propanal, hexanal, and 4-hydroxynonenal (4-HNE). These compounds contribute to peroxidative damage, notably in reproductive cells, decreasing cell viability (61). Testicular tissues are susceptible to oxidative stress due to their high amount of unsaturated fatty acids, rapid cell division, and high mitochondrial oxygen demand. Environmental contaminants, nutrition, and exposure to X-rays and chemotherapy can further worsen this damage (62).

Monosodium glutamate has been found to generate oxidative stress in experimental rats through chronic administration at doses of 4 mg/kg body weight and sub-chronic administration at dosages of 0.6 to 1.6 mg/kg body weight (63). Once ingested, MG is transformed into sodium ions and L-glutamate (2). The reproductive organs are rich in glutamate receptors, and when these receptors are overstimulated by glutamate or its analogs, it leads to increased ROS generation and lipid peroxidation (64).

Egbuonu et al. (65) stated that an increment in levels of MDA after MG treatment returns to oxidative stress. Ayoola et al. (66) also reported higher nitrite concentrations in experimental animals after MG administration, indicating oxidative stress. A decline in the activities of antioxidant enzymes such as CAT, SOD, GR, and GPx, likely due to the excellent production of ROS, was observed by Wang et al. (67). The inhibition of antioxidant enzymes after MG exposure may result from ROS protein inactivation (68).

This pre-administration of RUT and MOLE attenuated the effects generated by MG. The considerable reduction in oxidative stress markers (MDA, nitric oxide), augmentation of GSH level, and the restoration of antioxidant enzyme (SOD, CAT, GPx, and GR) activities imply that these substances boost the testicular antioxidant defense system.

FTIR spectrum of crude aqueous MOLE in this study showed the presence of various functional groups, including hydroxyl groups (OH), carbonyl groups (CO), alkanes (CH), and others. This outcome proposes that the extract contains water, fatty acids, lipids, proteins, polysaccharides, and maybe phenolic or aromatic compounds. The presence of these functional groups promotes the extract’s potential bioactive qualities, which are commonly connected to its medical and nutritional advantages.

*Moringa oleifera* leaves are rich in bioactive components, including vital minerals and phytonutrients (69). These necessary nutrients consist of proteins, vitamins (E, C, beta-carotene, and B6), minerals (such as calcium, phosphorus, and magnesium), and fatty acids (70). The bioactive phytonutrients in MO leaves include flavonoids (like quercetin and kaempferol) and phenolic acids such as ferulic, gallic, vanillic, and ellagic acids, with chlorogenic acid being the most abundant, as well as glycosides, saponins, alkaloids, tannins, isothiocyanates, and glucosinolates (71). Rutin (quercetin-3-O-rutin or rutinoside) is an abundant flavonoid component in numerous fruits and vegetables (17).

*Moringa oleifera* has continuously shown antioxidative properties through much research. It has been shown to increase glutathione concentrations in cells exposed to 250 μg/mL of the extract (36), reduce lipid peroxidation, and enhance antioxidant defenses by elevating activities of SOD, CAT, GSH, and ascorbic acid in the testes of diabetes mellitus-induced rats, suggesting a protective role in male fertility (72). Additionally, MO has been demonstrated to raise SOD and CAT activities while lowering MDA in animal models exposed to environmental toxicants (73). It also boosted testicular levels of GPx (74) and lowered glutathione S-transferase (GST) activity (75). In oxidative stress models, *Moringa* extract dramatically lowered MDA levels in acetaminophen-induced oxidative stress (76) and reduced MDA concentrations in obese rats while enhancing antioxidant enzymes like SOD, CAT, and GSH (40). Fakurazi et al. (76) further confirmed a substantial increase in catalase activity with *Moringa* extract treatment (*p* < 0.05).

Our results with RUT and MOLE coincide with recent findings revealing that some flavonoids from plants and fruits are potent O_2_•− scavengers (77). Moreover, various investigations have revealed that the pharmacological effects of flavonoids are connected to their antioxidant activity, which may entail scavenging OH• and O_2_•−, chelating metal ions, and exerting synergistic effects with other antioxidant metabolites (78). Quercetin is a dietary oxidant that boosts the antioxidant defense system and removes oxygen radicals created during cellular metabolism. The antioxidant activity of quercetin comprises various processes. On the one hand, as a polyphenol, the polyphenol hydroxyl group in its molecular structure can act as a hydrogen donor and combine with free radicals to directly scavenge ROS (60). Together with these findings, our results suggest that flavonoids could be major active components of RUT and MOLE in addition to phenolic chemicals in MOLE.

Organ weight analysis is a crucial marker in toxicological research for determining the detrimental effects of substances (79). Studies have revealed that changes in reproductive organ weight significantly indicate changes in reproductive hormone levels (80). Consistent with these findings, prior studies have demonstrated that the testicular weight of rats treated with MG considerably decreased (80, 81). Our findings supported this data since MG treatment resulted in a reduction in relative testicular weight. Additionally, we detected a significant drop in serum testosterone, LH, and FSH levels, which accords with findings from multiple other studies (80, 82). These results may be related to the reproductive system’s susceptibility to glutamate-induced damage due to many glutamate receptors in reproductive organs and sperm. Excitative toxicity damage to these cells was caused by excessive glutamate. Besides, glutamate-induced toxicity and elevated levels of ROS may interrupt hormonal balance by impairing the hypothalamic–pituitary-gonadal (HPG) axis, a critical system for controlling reproductive function (83). The disruption is linked to downregulating genes’ encoded enzymes participating in steroidogenesis, LH receptor activity, and signal transduction (84). Consequently, there is a reduction in the release of LH and FSH from the anterior pituitary gland (85). Decreased LH secretion hinders the stimulation of Leydig cells, leading to insufficient testosterone production (86), while lower FSH levels adversely influence the release of androgen-binding protein (ABP), which is important for concentrating testosterone (87). These hormonal disruptions are correlated to reduce spermatogenesis (82), which can negatively influence the development and preservation of testicular tissue, potentially reducing testis’ weight (43, 88). Since testis weight is correlated with the mass of differentiated spermatogenic cells, the detected decrease may be ascribed to a lower density of germ cells and mature spermatids (89).

Rutin’s structural closeness to estradiol significantly decreases toxicity induced by Zearalenone (ZEN). RUT has the ability to competitively bind to estrogen receptors, hence decreasing the deleterious effects of ZEN on these receptors. This competitive binding may help manage the hormonal abnormalities generated by ZEN exposure, notably throughout the reproductive system (90). Elsawy et al. (25) established RUT’s potential to restore lowered serum levels of testosterone (T), LH, and FSH in male mice exposed to CCl4, attributing this impact to its antioxidant characteristics. Consistent with these observations, Sayed et al. (90) found that RUT successfully ameliorated endocrine abnormalities generated by ZEN. MOLE at 300 mg/kg has been proven to raise serum testosterone, FSH, LH, sperm count, and sperm motility. The androgenic effects of MOLE on the male reproductive system have been demonstrated *in vitro* (36). Studies on male rats and rabbit bucks have indicated that MOLE dramatically enhances serum testosterone levels and the gene expression of LH and FSH (75, 91).

In this work, MG treatment promoted apoptosis in testicular germ cells, as demonstrated by increased expression of pro-apoptotic markers Bax and caspase-3, alongside decreased expression of the anti-apoptotic protein Bcl-2. A positive immunoreactivity of caspase-3 in the germinal epithelium and Leydig cells indicated the elevation of apoptosis. These results are reliable with those of Anbarkeh et al. (88), who noted that exposure to MG-activated caspase-3, a key enzyme in the apoptosis pathway, guides to increased germ cell apoptosis and subsequent testicular atrophy in mice. Similarly, Sarhan (92) found that MG promoted apoptosis in the testicular cells of rats.

In this investigation, rutin (a flavonoid constituent) and *Moringa oleifera* leaf extract and their flavonoid and polyphenolic fractions revealed strong anti-apoptotic protective effects against MG-induced apoptosis. This safeguard was proved by a decline in pro-apoptotic markers Bax and caspase-3 levels and increased anti-apoptotic protein Bcl-2 in testicular tissues. These results were also confirmed by a negative to moderate immunoreactivity of caspase-3 in the reproductive epithelium and Leydig cells.

NF-kB is known to stimulate the transcription of genes implicated in the apoptosis of male germ cells, often through the stimulation of Bax/Bcl-2 and the start of caspases (93). Furthermore, it has been shown that methods lowering ROS production ensure that the Bcl-2 gene prevents apoptosis, safeguarding the cells (94). *Moringa oleifera* extract downregulates caspase-3 and inhibits the activation of pathways such as NF-kB and phosphatidylinositide 3-kinase/protein kinase B (P13K/PKB), consequently inhibiting testicular apoptosis by reducing Bax expression (95). This action helps avoid male infertility. Sayed et al. (90) observed that RUT suppresses the apoptotic signaling cascade by enhancing the structural integrity of the spermatogenic tubules in the testes of mice exposed to Zearalenone (ZEN).

Our study indicated that rats exposed to MG displayed higher testicular levels of inflammatory markers, such as TNF-*α* and IL-1β. High COX-2 antibody seen in both the germinal epithelium and Leydig cells, further corroborating these effects’ heightened inflammation. These findings are comparable with those of Kumar et al. (96), who reported that the inflammatory responses initiated by MG would contribute to dysfunction and damage of the testis. The exposure to MG will induce oxidative stress, a precursor to inflammation (97). Oxidative stress may activate a transcription factor, “NF-κB” (98), which transfers to the nucleus, leading to the production of inflammatory mediators like IL-1β, IL-6 and TNF-α (99, 100).

When RUT and MOLE were given before the study, the levels of TNF-α and IL-1β in the testicles went down, which showed an anti-inflammatory response. This impact is further substantiated by the negative to moderate COX-2 antibody detected in the germinal epithelium and Leydig cells. MOLE has shown the capacity to lower the production of pro-inflammatory cytokines, including TNF-α and IL-6, and suppress the expression of RelA, a gene implicated in NF-κB p65 activation during inflammation. This outcome correlates with recent findings on the anti-inflammatory properties of *Moringa oleifera* (101, 102). Moreover, RUT from *Marrubium alysson* L. has shown potent anti-inflammatory efficacy in protecting testicular functioning in methotrexate-injected mice (103).

Our study suggests that MG treatment generates considerable oxidative stress, hormonal abnormalities, apoptosis, and inflammation, resulting in widespread cellular damage and testicular toxicity. The histological, histochemical, and immunohistochemical examinations of the testes confirmed these pathological effects, which appeared as pyknosis and reduced the heights of the germinal epithelium. Degenerated seminiferous tubules with few or no sperms and detached spermatogenic cells from the basement membranes with desquamated cells appeared. Interstitial edema, vacuolation, inflammatory infiltration and congested blood vessels were shown. Significant depositions of collagen fibers in capsules, interstitial and basal lamina of seminiferous tubules exhibited severity of damage. Strong PAS reaction shown in the basal lamina of tubules, spermatogenic cells and interstitial tissues detects structural and functional disorders of testes. The decline in cell proliferation appeared as most seminiferous tubules containing few or no sperm confirmed through a weak PCNA immunoreaction in germinal epithelium.

Due to its propensity to influence reproductive cells, MG can cause sperm modifications, histological changes, hormonal imbalances, and oxidative damage, finally leading to problems in reproductive function (104). Histological changes in the reproductive organs have been documented in rats treated with MG, including detachment and vacuolation of the seminal epithelium, degeneration of spermatogenesis, and edema inside the testicular lumen (80). Exfoliation of spermatocytes and spermatids within the lumens of seminiferous tubules was shown after MG treatment (105). Many spermatogenic cells appeared necrotic, with pyknotic nuclei and dilated, congested blood vessels shown under MG’s effects on the testes (106).

Severe histological changes such as degeneration of seminiferous tubules and reduction of sperm production observed by Acikel-Elmas et al. (107) resulted in oxidative stress and apoptosis produced after high doses of exposure MG. Abdelhameed et al. (103) stressed that oxidative stress and inflammation are significant factors in testicular injury, with elevated ROS levels destroying cellular components such as lipids, proteins, and DNA, ultimately compromising testicular function (108). Moreover, degenerative alterations in the seminiferous tubules produced by MG lead to decreased spermatogenesis and lower overall testis weight (83, 109).

The ameliorative effects of rutin and *Moringa oleifera* leaf extract in this study were demonstrated by noticeable improvements of histopathological changes in testicular sections returned to their constituents. Flavonoids have antioxidant and anti-inflammatory characteristics and can potentially improve the toxicity of testes induced by Zearalenone (110). The antioxidant property of rutin in reducing reproductive toxicity of germinal epithelium, cellular configuration and structural integrity in mice induced by cadmium was emphasized by Abarikwu et al. (111). Similar protective effects of RUT against testicular toxicity generated by several substances have been demonstrated in male mice (90, 111).

*Moringa oleifera* extract contains antioxidative, anti-inflammatory, anti-diabetic, anti-obesity, and anti-apoptotic activities (40, 101). Studies have demonstrated that *Moringa* treatment improves testicular shape, boosts spermatogenic cell activity, and raises sperm density in numerous animal models. In rabbit bucks, large dosages of *Moringa* leaves led to increased germinal cell activity, improved spermatozoa presence in the lumen of seminiferous tubules, and more significant interstitial regions with normal Leydig cells compared to non-supplemented bucks (39, 112). Additionally, treatment with *Moringa* in male rats dramatically enhanced seminiferous tubule width, epithelial height, spermatogenic efficiency, and the number of Sertoli and spermatogenic cells (37). MO also demonstrated ameliorative effects on the testicular histology of diabetic rats, expanding the number of spermatogonia and spermatids, increasing seminiferous tubule diameter and Leydig cell nuclear diameter, and boosting epididymal weight (72, 113).

Study limitation includes no direct clinical trials on humans in this concern, hence further clinical studies are needed.

## 5 Conclusion

The ameliorative effects of rutin and *Moringa oleifera* leaf extract against monosodium glutamate-induced testicular toxicity in rats were emphasized in this study and can be attributed to their potent antioxidant, anti-inflammatory, and anti-apoptotic characteristics. So, they may serve as a protective agent to alleviate reproductive toxicity caused by dietary and environmental toxins. Additional studies are required to explore the long-term effects, optimal dosage for therapeutic applications, and a more detailed exploration of their mechanism of action at the molecular level of these compounds.

## Data Availability

The original contributions presented in the study are included in the article/supplementary material, further inquiries can be directed to the corresponding author.

## Glossary

4-HNE: 4-hydroxynonenal
ANOVA: Analysis of Variance
Bax: B-cell lymphoma protein 2 (Bcl-2)-associated X
Bcl-2: B-cell lymphoma protein 2
CAGR: Compound Annual Growth Rate
CAT: Catalase
CONT: Control
COX-2: Cyclooxygenase-2
ELISA: Enzyme-linked immunosorbent assay
FSH: Follicle-stimulating hormone
FTIR: Fourier transform infrared
GPx: Glutathione peroxidase
GR: Glutathione reductase
GSH: Reduced glutathione
H&E: Hematoxylin and Eosin
HPG: Hypothalamic–pituitary-gonadal
IL-1β: Interleukin-1 beta
LD_50_: Lethal dose 50
LH: Luteinizing hormone
LOOHs: lipid hydroperoxides
LPO: Lipid peroxide
MAPK: Mitogen-activated protein kinase
MDA: Malondialdehyde
mL: Milliliter
MG: Monosodium glutamate
MO: *Moringa oleifera*
MOLE: *Moringa oleifera* leaf extract
mRNA: Messenger ribonucleic acid
NF-κB: Nuclear factor-kappa B
NO: Nitric oxide
Nrf2: Nuclear factor erythroid 2–related factor 2
OS: Oxidative stress
P13K/PKB: Phosphatidylinositide 3-kinase/protein kinase B
PAS: Periodic acid-Schiff
PCNA: Proliferating cell nuclear antigen
ROS: Reactive oxygen species
RUT: Rutin
RWT: Relative testicular weight
SOD: Superoxide dismutase
TNF-α: Tumor necrosis factor-alpha
USD: United States dollar
ZEN: Zearalenone
